# Quantifying the confounding effect of pigmentation on measured skin tissue optical properties: a comparison of colorimetry with spatial frequency domain imaging

**DOI:** 10.1117/1.JBO.27.3.036002

**Published:** 2022-03-23

**Authors:** Thinh Phan, Rebecca Rowland, Adrien Ponticorvo, Binh Cong Le, Seyed A. Sharif, Gordon T. Kennedy, Robert H. Wilson, Anthony J. Durkin

**Affiliations:** a University of California, Irvine, Beckman Laser Institute and Medical Clinic, Irvine, California, United States; b University of California, Irvine, Department of Medicine, Irvine, California, United States; c University of California, Irvine, Health Policy Research Institute, Irvine, California, United States; d University of California, Irvine, Department of Biomedical Engineering, Irvine, California, United States

**Keywords:** spatial frequency domain imaging, colorimeter, melanin, scattering coefficient, absorption coefficient, skin, epidermis, pigmentation, multispectral imaging

## Abstract

**Significance:**

Spatial frequency domain imaging (SFDI) is a wide-field diffuse optical imaging technique for separately quantifying tissue reduced scattering (${\mu_{s}}^{\prime }$) and absorption ($\mu_{a}$) coefficients at multiple wavelengths, providing wide potential utility for clinical applications such as burn wound characterization and cancer detection. However, measured ${\mu_{s}}^{\prime }$ and $\mu_{a}$ can be confounded by absorption from melanin in patients with highly pigmented skin. This issue arises because epidermal melanin is highly absorbing for visible wavelengths and standard homogeneous light–tissue interaction models do not properly account for this complexity. Tristimulus colorimetry (which quantifies pigmentation using the L* “lightness” parameter) can provide a point of comparison between $\mu_{a}$, ${\mu_{s}}^{\prime }$, and skin pigmentation.

**Aim:**

We systematically compare SFDI and colorimetry parameters to quantify confounding effects of pigmentation on measured skin ${\mu_{s}}^{\prime }$ and $\mu_{a}$. We assess the correlation between SFDI and colorimetry parameters as a function of wavelength.

**Approach:**

${\mu_{s}}^{\prime }$ and $\mu_{a}$ from the palm and ventral forearm were measured for 15 healthy subjects with a wide range of skin pigmentation levels (Fitzpatrick types I to VI) using a Reflect RS^®^ (Modulim, Inc., Irvine, California) SFDI instrument (eight wavelengths, 471 to 851 nm). L* was measured using a Chroma Meter CR-400 (Konica Minolta Sensing, Inc., Tokyo). Linear correlation coefficients were calculated between L* and ${\mu_{s}}^{\prime }$ and between L* and $\mu_{a}$ at all wavelengths.

**Results:**

For the ventral forearm, strong linear correlations between measured L* and ${\mu_{s}}^{\prime }$ values were observed at shorter wavelengths (R>0.92 at ≤659  nm), where absorption from melanin confounded the measured ${\mu_{s}}^{\prime }$. These correlations were weaker for the palm (R<0.59 at ≤659  nm), which has less melanin than the forearm. Similar relationships were observed between L* and $\mu_{a}$.

**Conclusions:**

We quantified the effects of epidermal melanin on skin ${\mu_{s}}^{\prime }$ and $\mu_{a}$ measured with SFDI. This information may help characterize and correct pigmentation-related inaccuracies in SFDI skin measurements.

## 1 Introduction

Skin color is a major area of study in dermatology, and more broadly, renewed emphasis has recently been placed on identifying disparities in accuracy of clinical technologies such as pulse oximeters among patients with different skin tones.^1^^–^^4^ Pigmentation elements, such as melanin, hemoglobin, and carotenoids, selectively absorb light at specific wavelengths; the remainder of the light can be scattered back to the surface to be detected by the eye as a “skin color.” This detected “color” is a combination of the spectrum of the incident light, the specular and diffuse reflectance of the tissue, and the spectral response of the eye. Colorimetric measurements of the skin, which rely on diffusely backscattered light, can provide information about the relative amounts of these absorbing components in the skin. The use of skin colorimetry has been common for the past 50 years to characterize reactivity of normal skin to light (e.g., tanning and erythema) and to diagnose and monitor conditions, such as port-wine stain and skin cancer.^2^

In dermatology, tristimulus colorimeters collect reflectance data within the visible spectrum and use the International Commission on Illumination (CIE) L*a*b* color space for quantitative skin color classification.^5^^,^^6^ Here, L* is used to describe lightness, a* is the green to red component, and b* is the blue to yellow component. In contrast to the Fitzpatrick scale, which is a subjective, survey-based measure of skin’s potential response to UV radiation,^5^ the L* value has been used to help characterize pigmentation level on a more objective physiological scale,^7^ providing a surrogate measurement for melanin content.

Recently, spatial frequency domain imaging (SFDI) has emerged as a more quantitative, functional imaging technique for skin,^8^^–^^10^ demonstrating strong potential for clinical applications including burn wound assessment^11^ and cancer detection.^12^ SFDI measures the tissue absorption ($\mu_{a}$) and reduced scattering (${\mu_{s}}^{\prime }$) coefficients at each wavelength by projecting spatially modulated patterns onto the tissue surface and detecting the backscattered light with a camera. A mathematical model of light–tissue interaction is then used to separate and quantify the effects of tissue absorption ($\mu_{a}$ due to chromophores such as hemoglobin and melanin) from the effects of tissue scattering (${\mu_{s}}^{\prime }$, attributed to morphology of intracellular and extracellular tissue components such as organelles and collagen fibers) from the measured diffuse reflectance as a function of spatial frequency.^8^^,^^10^^,^^13^^–^^15^

However, a recent publication from our group showed that ${\mu_{s}}^{\prime }$ values measured with SFDI using a homogeneous model of skin were unphysically lower at visible wavelengths (450 to 650 nm) than near-infrared (NIR) wavelengths for subjects with darker skin (e.g., Fitzpatrick skin types V and VI),^16^ even though melanin is not expected to have a significant effect on tissue scattering.^15^^,^^17^ In that report, the measured scattering spectrum ranging from visible to NIR wavelengths did not follow the inverse-power-law pattern typical of Rayleigh and Mie scattering theory. Because the subjects’ skin types in this previous study were quantified using a subjective Fitzpatrick skin scale survey, it is important to develop an improved quantitative understanding of this phenomenon by characterizing the relationship between SFDI and colorimetry data for patients with a wide range of skin types.

In this study, we perform pairwise correlation analyses between L* (measured with a tristimulus colorimeter) and $\mu_{a}$ and ${\mu_{s}}^{\prime }$ (measured with a commercially available SFDI device). At visible wavelengths, this analysis highlights a strong linear correlation between L* and each of $\mu_{a}$ and ${\mu_{s}}^{\prime }$ in subjects with dark skin. However, the correlation between L* and ${\mu_{s}}^{\prime }$ becomes insignificant for NIR wavelengths (which are not strongly absorbed by melanin) and for the palm (which is expected to have at least a factor of 2 less melanin content than the forearm in subjects with Fitzpatrick skin types IV and V).^18^ These results indicate that SFDI measurements of skin can be strongly confounded by melanin, making the performance of SFDI very similar to that of a standard tristimulus colorimeter when the measured tissue is melanin-rich. Therefore, for SFDI to provide accurate values of $\mu_{a}$ and ${\mu_{s}}^{\prime }$ at visible wavelengths for patients with dark skin, the technique must be modified to include direct quantification of melanin in the epidermis.

## 2 Methodology

### 2.1 Subject Enrollment

Fifteen subjects from 20 to 51 years old with no known dermatological complications were enrolled in the study. Subjects were recruited according to the University of California, Irvine’s Institutional Review Board protocol #2011-8370, and informed consent was obtained from all subjects. A breakdown of subject age, gender, and L* for the ventral forearm and palm is shown in Table 1.

**Table 1:** Subject age, gender, and L* values obtained from the ventral forearm and palm. The order was arranged according to increasing L* values of the ventral forearm.

Subject number (in records)	Age	Gender	L* ventral forearm	L* palm
1	21	M	36.74	52.53
2	20	F	39.14	60.97
3	21	F	42.26	62.2
4	21	M	43.89	56.93
5	22	F	46.24	54.28
6	40	M	56.22	62.99
7	27	M	56.96	62.87
8	29	M	57.69	54.61
9	26	F	59.85	65.95
10	31	M	61.5	65.78
11	28	M	62.07	59.40
12	23	M	63.35	59.68
13	51	M	63.48	61.42
14	25	F	63.74	67.87
15	42	F	66.38	64.89

### 2.2 Measuring L* with Colorimetry

**Fig. 1:**
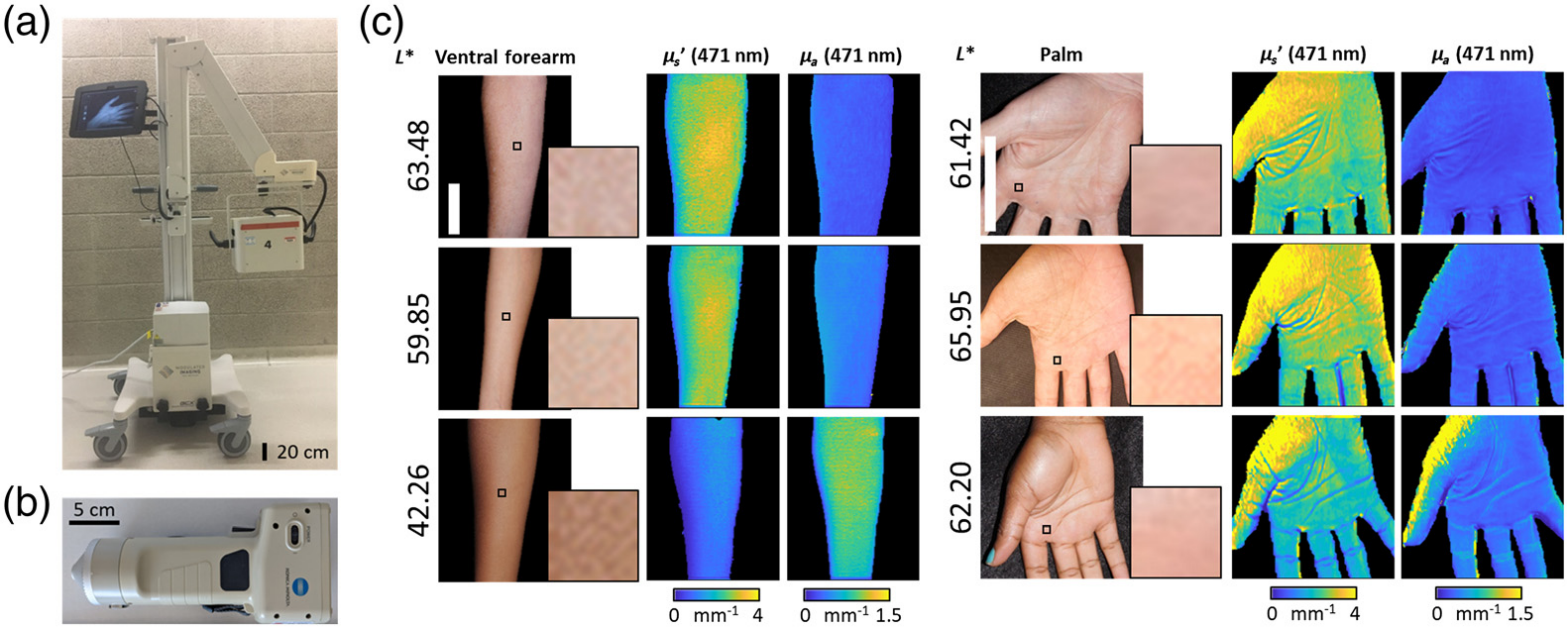
(a) SFDI instrument, Reflect RS^®^ (Modulim, Inc., Irvine, California). (b) Colorimeter instrument, Chroma Meter CR-400 (Konica Minolta Sensing, Inc., Tokyo, Japan). (c) Example color images (with zoomed-in representative images), absorption coefficient maps, and reduced scattering maps of ventral forearms and palms from subjects with a range of pigmentation levels as indicated by the colorimetric L* values. Scale bars=5  cm.

Measurement of skin pigmentation at each anatomical region was obtained using a skin colorimeter that provided values in CIE L*a*b* color space (Chroma Meter CR-400, Konica Minolta Sensing, Inc., Tokyo) [Fig. 1(b)]. The colorimeter provided quantitative point measurements of skin color, an alternative to the more subjective, survey-based Fitzpatrick scale.^6^ Specifically, we used the L* parameter to quantify “lightness” of skin on a 0 to 100 scale (0 = darkest, 100 = lightest). This measured lightness has been shown to correlate with melanin concentration in previous reports.^6^^,^^7^

The L* values for the majority of subjects were notably higher on the palm than on the ventral forearm, an expected result because the ventral forearm is expected to have higher melanin content than the palm. There were four subjects who had slightly higher L* values on the ventral forearm than the palm, but all four of these subjects had very light skin tone (lightly pigmented), so they were expected to have nearly identical L* values on the ventral forearm and palm, as was observed.

### 2.3 Measuring $\mu_{a}$ and ${\mu_{s}}^{\prime }$ with Spatial Frequency Domain Imaging

SFDI measurements were performed with the Reflect RS^®^ (Modulim, Inc., Irvine, California), a commercially available, cart-based, research grade system [Fig. 1(a)]. The device uses eight light-emitting diodes (LEDs) at visible to NIR wavelengths (471, 526, 591, 621, 659, 731, and 851 nm) to project spatially modulated sinusoidal patterns with multiple spatial frequencies (0 to $0.2\;{\mathrm{mm}}^{-1}$ in steps of $0.05\;{\mathrm{mm}}^{-1}$). Diffuse reflectance images are collected sequentially for each combination of wavelength and spatial frequency pattern and calibrated using a silicone-based tissue-simulating “phantom” with homogeneous known optical properties. The reflectance values are converted into optical property maps of the reduced scattering coefficient (${\mu_{s}}^{\prime }$) [Fig. 1(c)] and the absorption coefficient ($\mu_{a}$) using the MI Analysis (Modulim, Inc., Irvine, California) suite. This method for analysis is based on the Monte Carlo forward model that considers the imaged tissue to be a semi-infinite, homogeneous entity, as described previously.^9^^,^^10^^,^^16^ All further analysis, including region of interest (ROI) selection and statistical evaluation, was performed in MATLAB (R2020a, MathWorks, Natick, Massachusetts). For each optical property map, we chose a 40×40  pixel ($∼1\;{\mathrm{cm}}^{2}$) ROI to avoid regions that are susceptible to artifacts from abrupt changes of curvature. It is important to note that the ROI sampled by the colorimeter was similar in size to that sampled by the SFDI device.

### 2.4 Assessing Correlations between L* and SFDI Parameters

The relationship between L* and measured ${\mu_{s}}^{\prime }$ and between L* and measured $\mu_{a}$ was characterized using linear correlations. For each subject, the correlation coefficient R was calculated for the relationship between the measured L* value and the measured ${\mu_{s}}^{\prime }$ (and $\mu_{a}$) at each wavelength.

## 3 Results

### 3.1 Distribution of L* Values Measured with Tristimulus Colorimeter

**Fig. 2:**
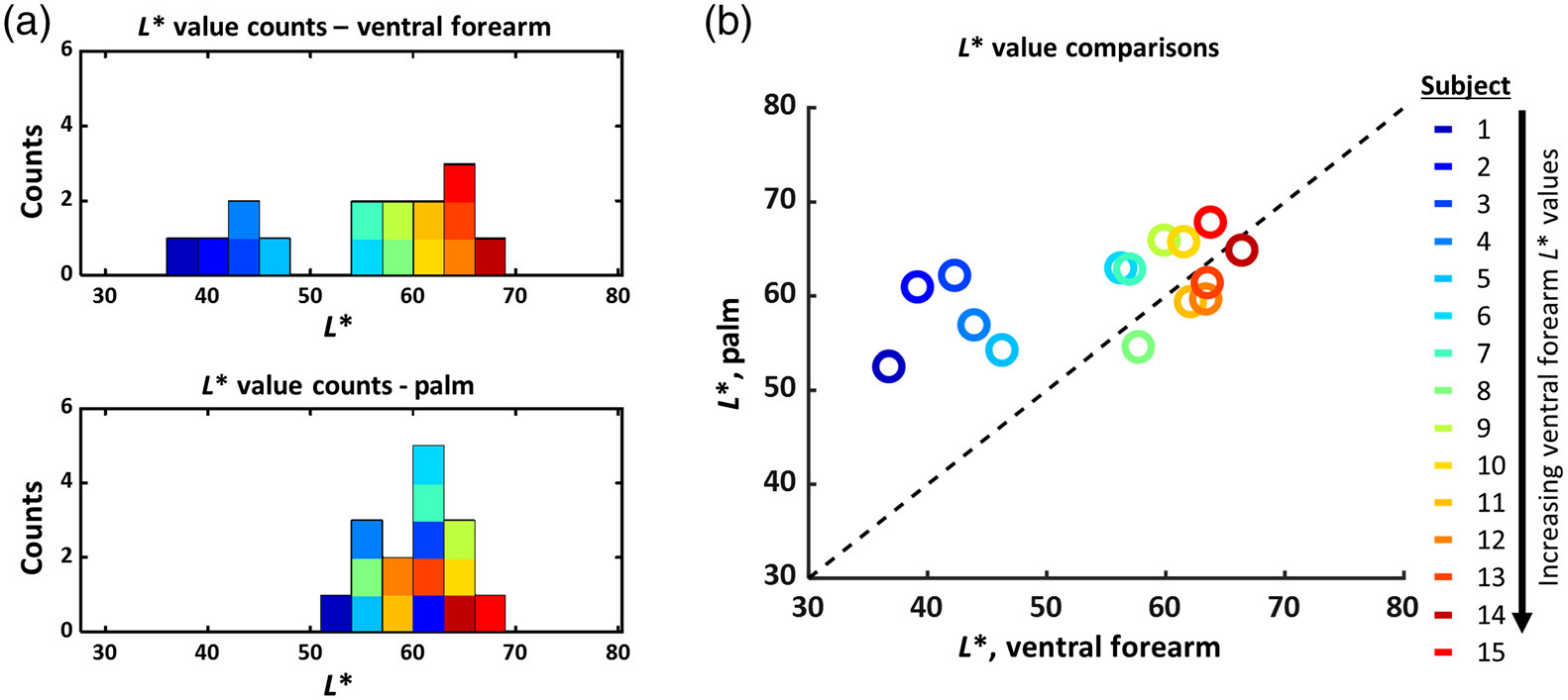
(a) Distribution of L* values for all subjects, measured in the ventral forearm and palm. The ventral forearm L* values follow a bimodal distribution (L*<50 represents patients with darker skin), whereas the palm values follow a unimodal distribution. The color scale is arranged by ventral forearm L* value, where blue is low L* (dark skin) and red is high L* (light skin). This color scheme is repeated throughout this report. (b) The L* values for the ventral forearm and palm are nearly identical for patients with lighter skin, but the L* values of the palm are systematically higher than those of the ventral forearm for patients with dark skin.

The distributions of L* values measured with the colorimeter on the palm and ventral forearm are shown in Figs. 2(a) and 2(b). The distribution of L* values in the palm shows little spread (mean: 60.8, standard deviation: 4.6), whereas the distribution of L* values measured from the ventral forearm has a larger spread (mean: 54.6, standard deviation: 10.1). The ventral forearm shows a bimodal distribution in L* values between subjects (where L*<50 corresponds to darker skin types), but this distribution is unimodal for the palm L* values. The dotted “y=x” line in Fig. 2(b) is shown as a reference. We expect that subjects with lighter skin tones would have L* values that lie closer to this line and that subjects with darker skin tones would have L* values that lie farther away from this line. These expected trends are indeed what is seen in Fig. 2(b).

### 3.2 Effects of Skin Pigmentation on SFDI-Measured ${\mu_{s}}^{\prime }$ and $\mu_{a}$

**Fig. 3:**
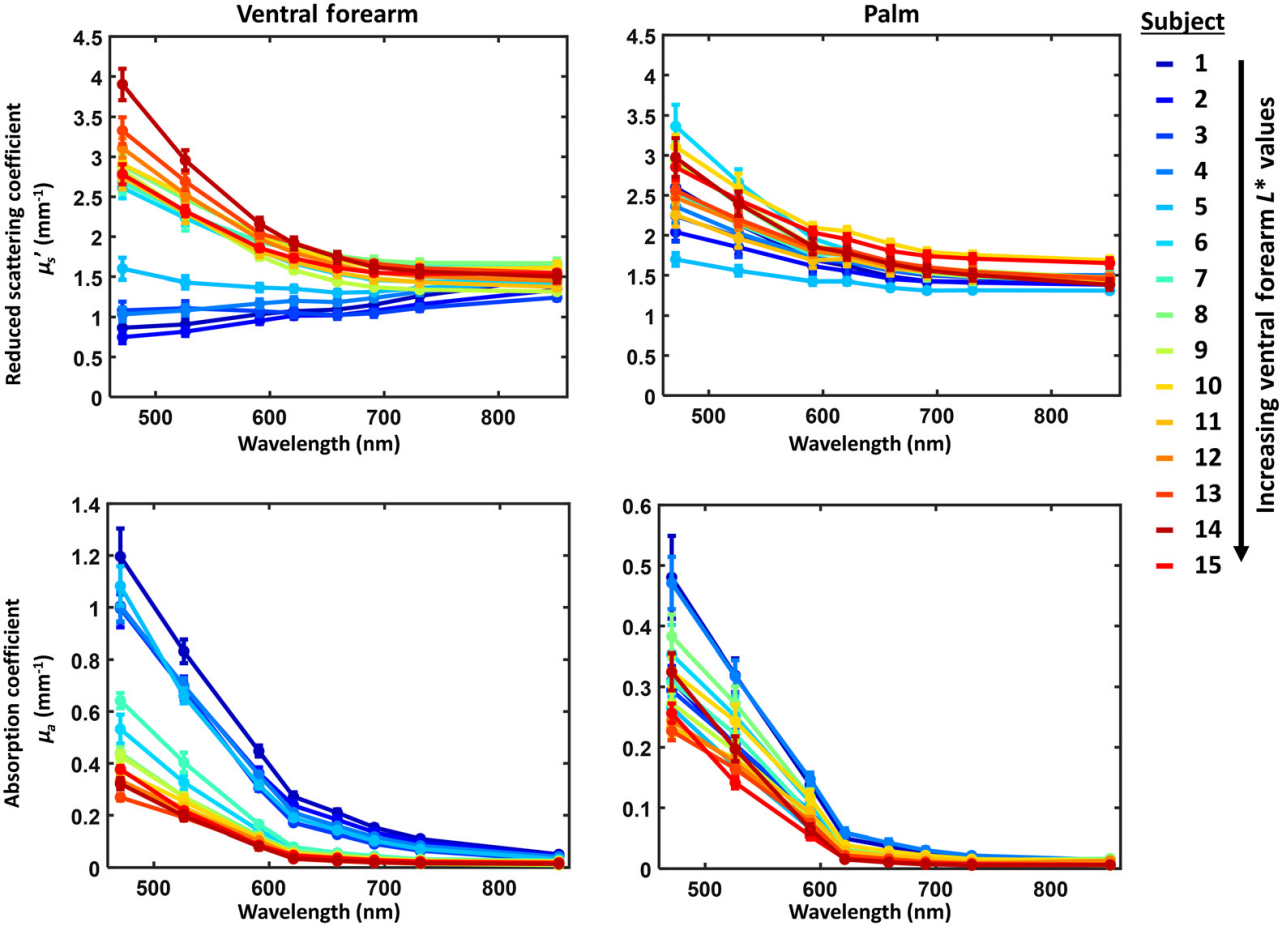
(Top) Reduced scattering and (bottom) absorption coefficients measured on (left) the palm and (right) ventral forearm measured with SFDI, using a homogeneous skin model. Each data point represents the mean (± standard deviation) of reduced scattering or absorption coefficients measured within the chosen ROIs. The color scales are arranged by ventral forearm L* value, where blue is low L* (dark skin) and red is high L* (light skin).

Figure 3 shows ventral forearm and palm measured ${\mu_{s}}^{\prime }$ and $\mu_{a}$ values at all wavelengths. The values reported here represent the mean (± standard deviation) values of the optical property of interest measured within the selected ROI. The measured ${\mu_{s}}^{\prime }$ and $\mu_{a}$ values for the palm show similar trends for all subjects, whereas the ventral forearm measurements vary notably between subjects with light skin (L*>50) and subjects with dark skin (L*<50). Subjects with L*<50 have measured ${\mu_{s}}^{\prime }$ spectra that do not follow the expected inverse power law distribution for tissue scattering.

**Fig. 4:**
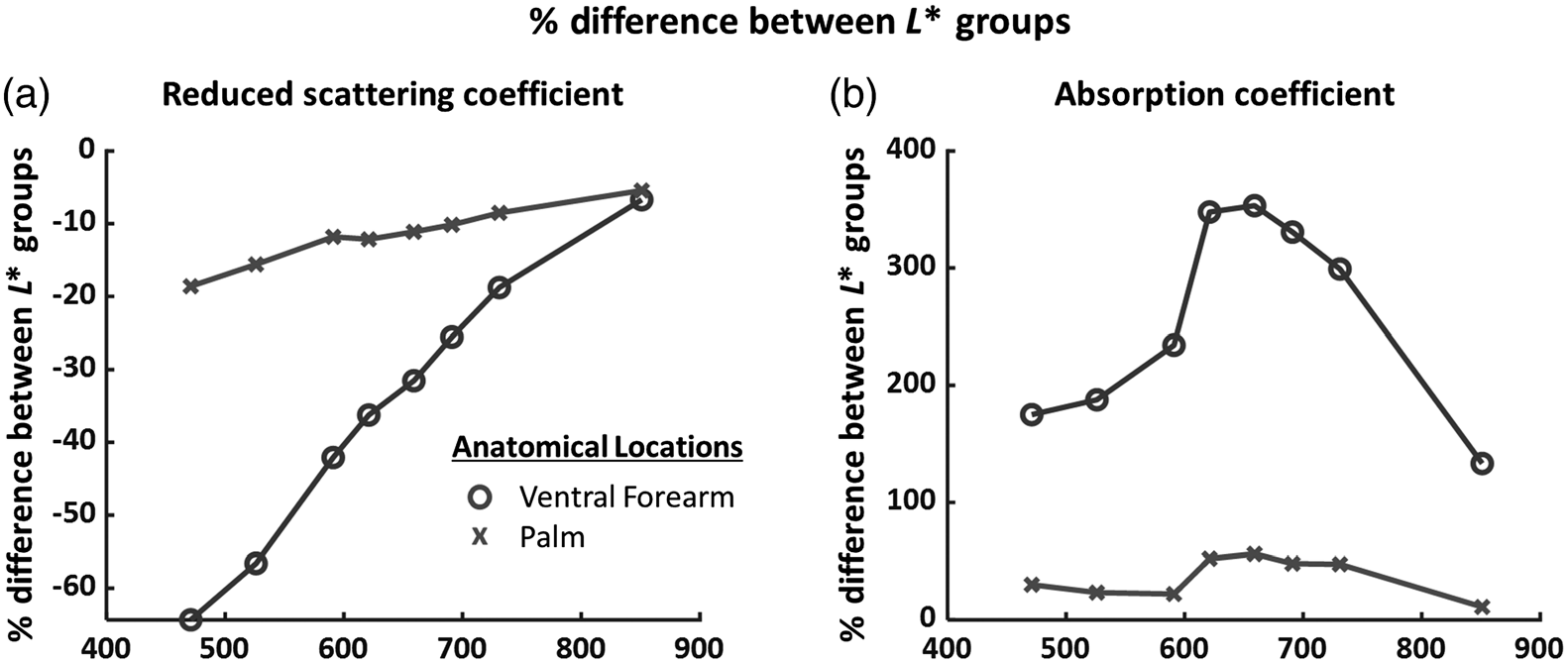
Mean percent difference in measured (a) ${\mu_{s}}^{\prime }$ values and (b) $\mu_{a}$ values between subjects with light skin (ventral forearm L*>50, n=10) and subjects with dark skin (ventral forearm L*<50, n=5).

Figure 4 shows the percentage difference between subjects with darker skin (N=5; L*<50) versus lighter skin (N=10; L*>50) for measured ${\mu_{s}}^{\prime }$ and $\mu_{a}$ at each wavelength. For the ventral forearm, subjects with L*<50 had measured ${\mu_{s}}^{\prime }$ values at 471 nm that were ∼65% lower than subjects with L*>50. For the palm, scattering in the darker skin (L*<50) group was only 20% less than the lighter skin (L*>50) group at 471 nm. Differences in measured ${\mu_{s}}^{\prime }$ between the two L* groupings decreased with increasing wavelength, and at 851 nm there was only a 10% difference in measured ${\mu_{s}}^{\prime }$ between the two groups for both the ventral forearm and palm. For the ventral forearm, the measured $\mu_{a}$ values at all wavelengths were at least 100% higher for the darker skin L* group than the lighter skin L* group, as expected. For the palm, where the melanin content is much lower than the ventral forearm, the measured $\mu_{a}$ values of the two L* groups were much more similar to each other (within ∼50% of each other at the shortest wavelengths).

**Fig. 5:**
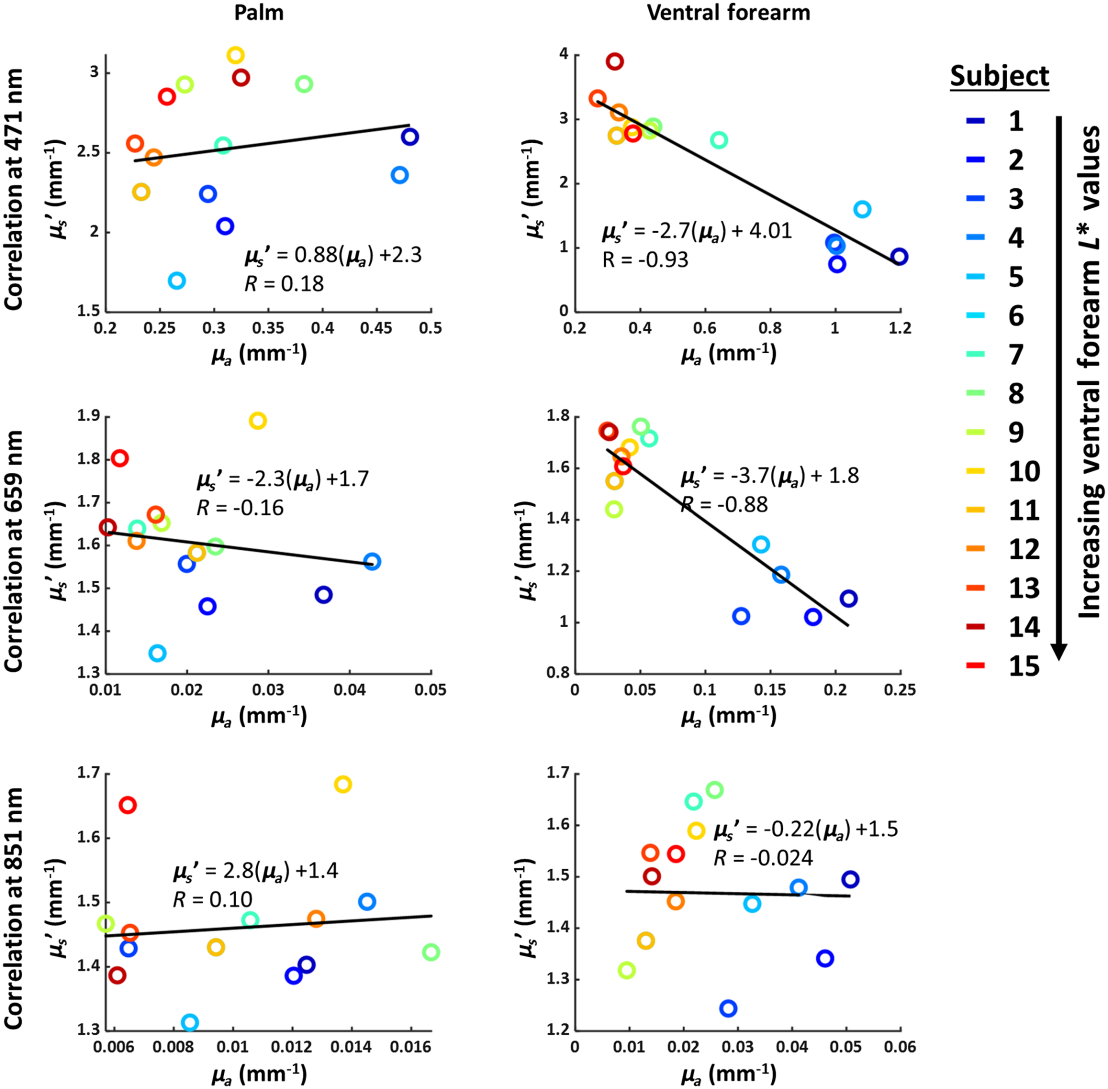
Correlations between measured absorption ($\mu_{a}$) and reduced scattering (${\mu_{s}}^{\prime }$) coefficients for the palm and ventral forearm at 471, 659, and 851 nm. The palm does not exhibit correlation between these parameters, but the ventral forearm exhibits very strong negative correlation between these two parameters at visible wavelengths.

Figure 5 shows correlations between measured $\mu_{a}$ and ${\mu_{s}}^{\prime }$ at 471, 659, and 851 nm for the ventral forearm and palm. The palm, which has very little pigmentation, exhibits no correlation between measured $\mu_{a}$ and ${\mu_{s}}^{\prime }$ at any measured wavelength (|R|<0.19). By contrast, the ventral forearm, which has high pigmentation levels in subjects with dark skin, does exhibit a strong negative correlation between measured $\mu_{a}$ and ${\mu_{s}}^{\prime }$ in the visible regime (R=−0.93 at 471 nm; R=−0.88 at 659 nm). However, this correlation does not persist at 851 nm (R=−0.024).

**Fig. 6:**
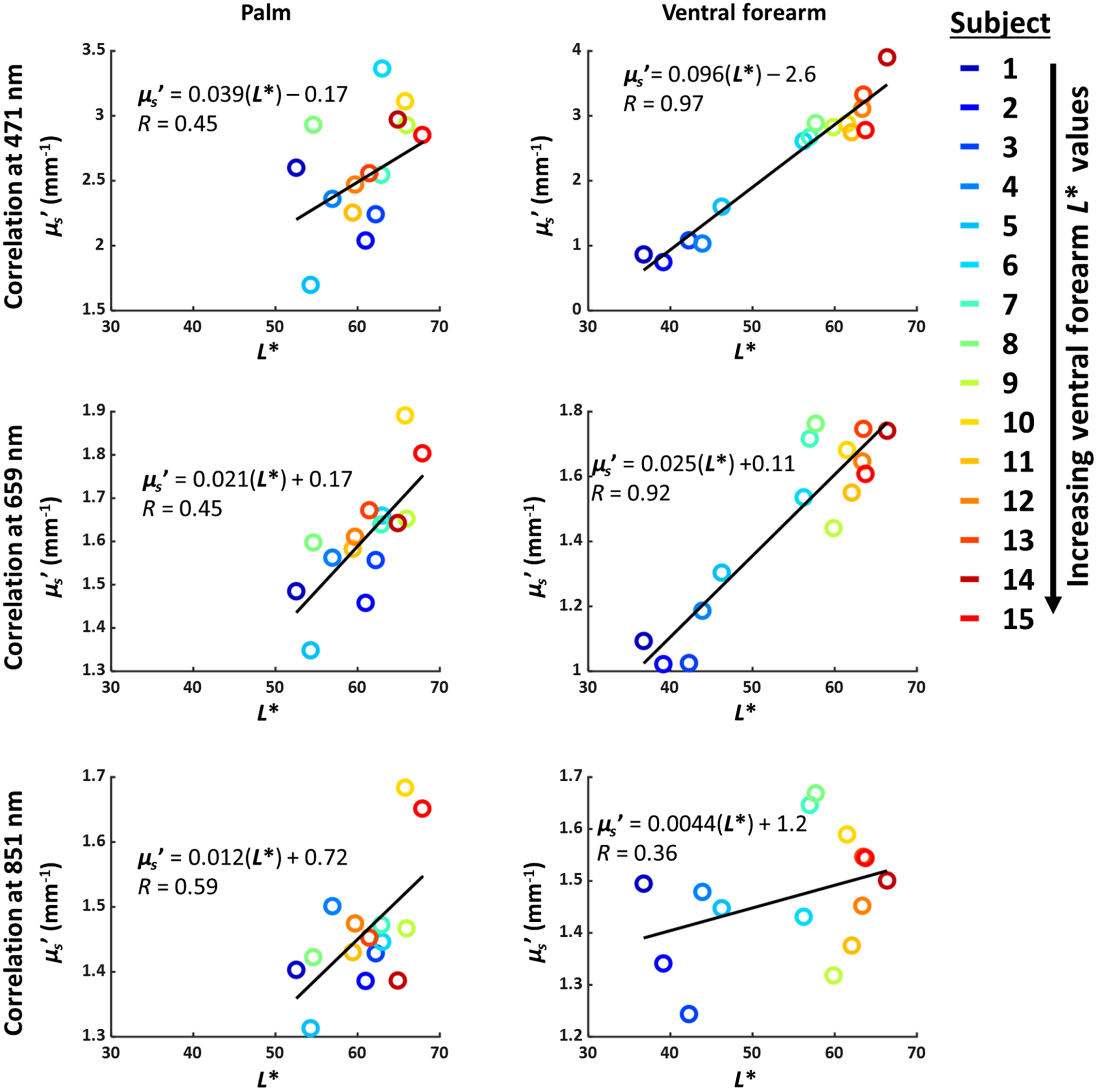
Correlations between L* (measured with colorimeter) and ${\mu_{s}}^{\prime }$ (measured with SFDI) for the palm and ventral forearm. The best-fit line to each set of data points is shown in black. For the ventral forearm, where patients with dark skin have high pigmentation, there is a very strong correlation between L* and measured ${\mu_{s}}^{\prime }$ at 471 and 659 nm, but this correlation vanishes at 851 nm and is not as strong for the palm, which has less pigmentation. This result suggests that the measured ${\mu_{s}}^{\prime }$ is being confounded by the presence of high pigmentation levels (low L*).

Figure 6 shows the relationship between L* and measured ${\mu_{s}}^{\prime }$ for the palm and ventral forearm. For the palm, there is a weak positive linear correlation (R<0.6) between L* and measured ${\mu_{s}}^{\prime }$. However, in the ventral forearm, there is a very strong positive correlation between L* and measured ${\mu_{s}}^{\prime }$ at 471 nm (R=0.97) and 659 nm (R=0.92). Lower L* values correspond to darker skin tone, so the positive correlation between L* and measured ${\mu_{s}}^{\prime }$ at visible wavelengths implies that tissue scattering values measured with SFDI are underestimated for patients with darker skin.

**Fig. 7:**
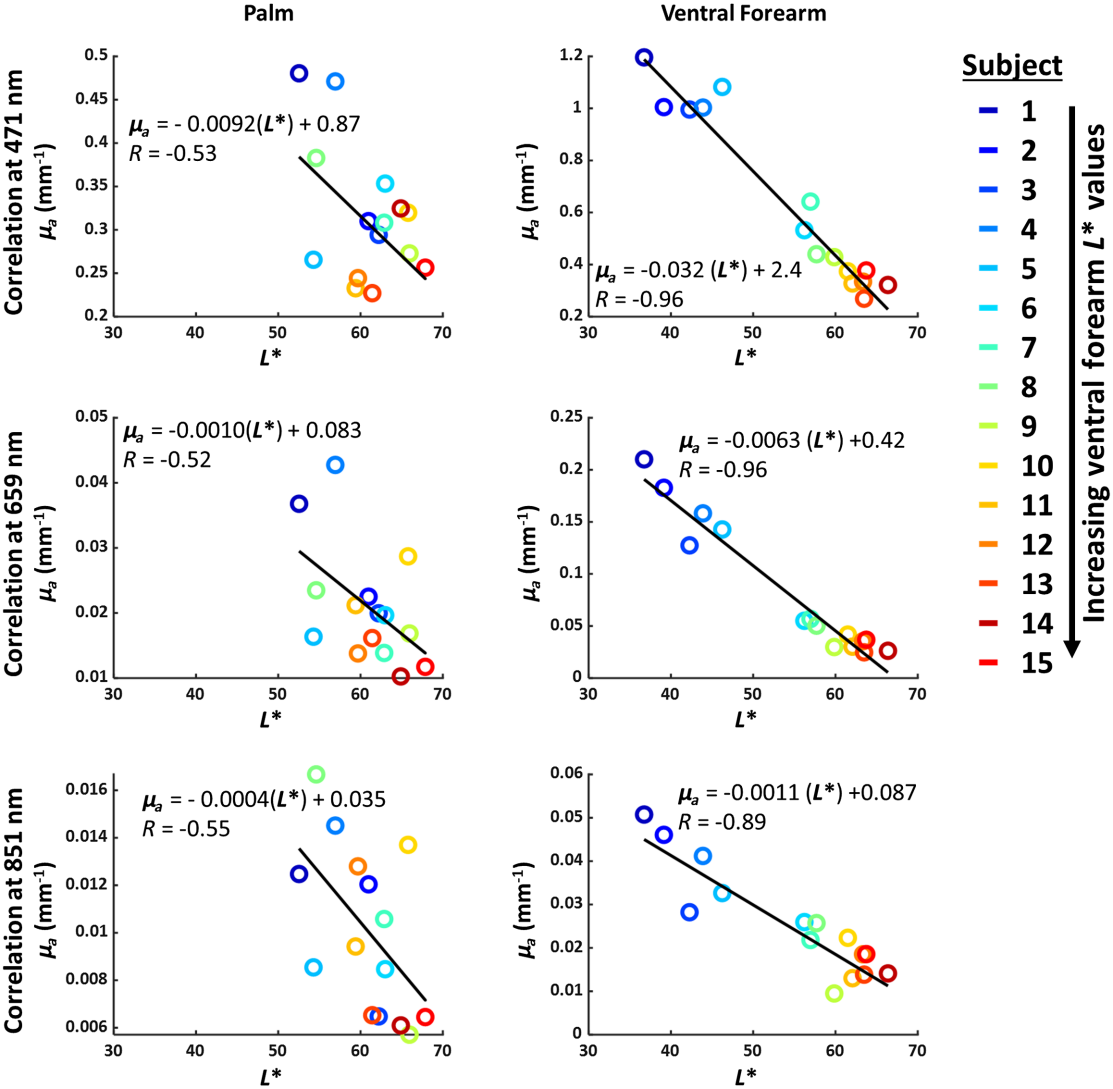
Correlations between L* (measured with colorimeter) and $\mu_{a}$ (measured with SFDI) for the palm and ventral forearm. The best-fit line to each set of data points is shown in black. For the ventral forearm, where patients with dark skin have high pigmentation, there is a very strong correlation between L* and measured ${\mu_{s}}^{\prime }$ at 471, 659 and 851 nm. For the palm, which has less pigmentation, the correlation is weaker (|R|<0.56).

**Fig. 8:**
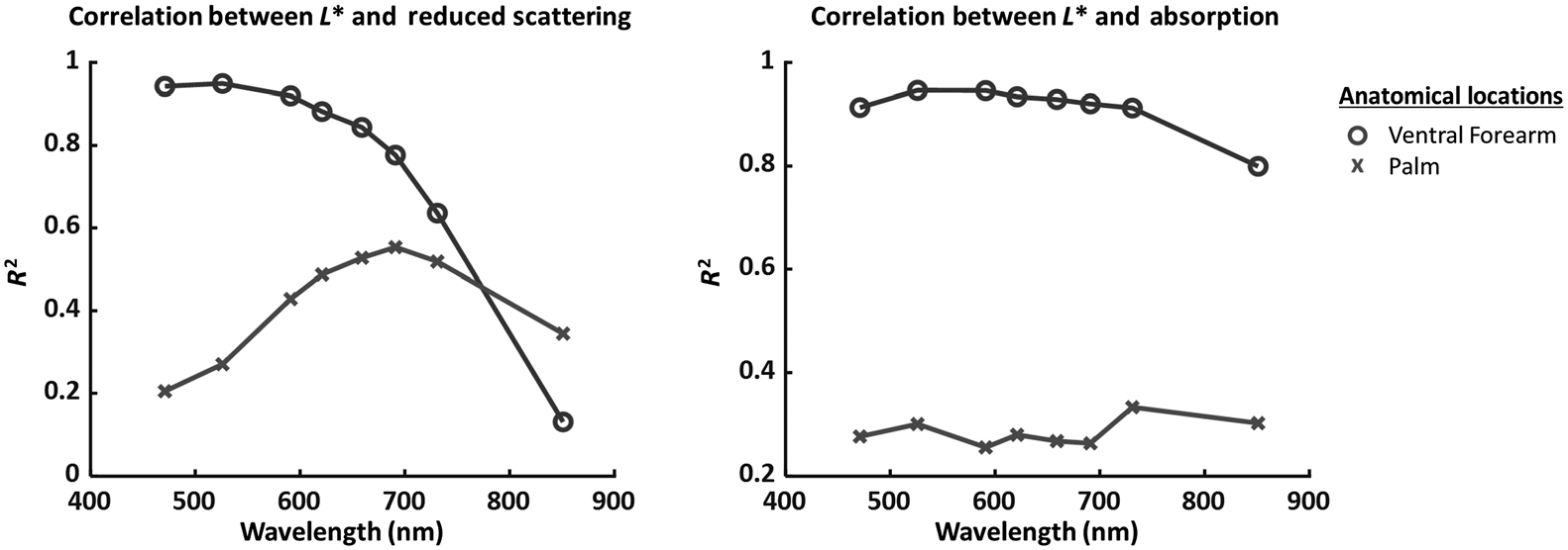
$R^{2}$ from linearly fitted L* and measured absorption ($\mu_{a}$) and reduced scattering (${\mu_{s}}^{\prime }$), plotted as a function of wavelength for the palm and ventral forearm.

Figure 7 shows the relationship between L* and measured $\mu_{a}$ for the palm and ventral forearm. Both the palm and ventral forearm show a negative correlation between L* and measured $\mu_{a}$. This correlation was much stronger in the ventral forearm (|R|>0.89) than in the palm (|R|<0.56). Figure 8 summarizes the correlations (quantified using $R^{2}$ values) between L* and the measured ${\mu_{s}}^{\prime }$ and $\mu_{a}$. Further analysis of the significance of each correlation is detailed in the accompanying supplementary section. Tables 2 and 3 detail the p-values for the linear fits between L* and each of measured ${\mu_{s}}^{\prime }$ and $\mu_{a}$, for each wavelength. For the ventral forearm, the correlations between L* and measured ${\mu_{s}}^{\prime }$ were significant for all wavelengths except 851 nm, and the correlations between L* and measured $\mu_{a}$ were significant at all wavelengths. For the palm, the correlations between L* and measured ${\mu_{s}}^{\prime }$ were significant from 591 to 731 nm, and the correlations between L* and measured $\mu_{a}$ were significant at all wavelengths.

**Table 2:** P-values for the linear fit between L* and reduced scattering for the palm and ventral forearm.

	471 nm	526 nm	591 nm	621 nm	659 nm	691 nm	731 nm	851 nm
Palm	0.090	0.047	0.0081	0.0038	0.0022	0.0015	0.0025	0.021
Ventral forearm	<0.0001	<0.0001	<0.0001	<0.0001	<0.0001	<0.0001	<0.0001	0.19

**Table 3:** P-values for the linear fit between L* and absorption for the palm and ventral forearm.

	471 nm	526 nm	591 nm	621 nm	659 nm	691 nm	731 nm	851 nm
Palm	0.044	0.035	0.055	0.043	0.049	0.050	0.024	0.034
Ventral forearm	<0.0001	<0.0001	<0.0001	<0.0001	<0.0001	<0.0001	<0.0001	<0.0001

## 4 Discussion

### 4.1 Comparison of Colorimetry and SFDI

In this report, we have shown a strong correlation between the melanin-dependent L* parameter and the SFDI-measured values of ${\mu_{s}}^{\prime }$ and $\mu_{a}$ in skin when a homogeneous light–tissue interaction model is used (Figs. 6 and 7). This effect is more pronounced at shorter (visible) wavelengths, where high absorption from epidermal melanin in subjects with darker skin greatly reduces the accuracy of the homogeneous model. We hypothesize that the measured reduced scattering values for subjects with light skin are accurate because epidermal absorption is not expected to confound the measured SFDI data for these subjects. As the L* value decreases further and further, the increase in epidermal melanin confounds the accuracy of the measured ${\mu_{s}}^{\prime }$ value more and more. Despite this obvious limitation, it is important to note that this correlation is not nearly as strong for NIR wavelengths (e.g., 851 nm), so a homogeneous skin model is likely a reasonable approximation in that regime, regardless of skin tone. This finding is corroborated by our recently-published study^16^ in which we showed that the ${\mu_{s}}^{\prime }$ values measured at 851 nm with SFDI clustered together for subjects with a wide range of skin tones but with the ${\mu_{s}}^{\prime }$ values for subjects with different skin tones deviated significantly from each other at visible wavelengths.

One key limitation of this study was that the L* measurements were only acquired from one representative spatial location from each body part studied (forearm and palm). Therefore, the absence of data on the spatial variability of L* could slightly affect the correlations between L* and the measured tissue absorption and scattering coefficients. However, these discrepancies are expected to be minor and not impactful on the overall results of the paper. This assumption is reasonable because the main finding of this study was the large differences between measured reduced scattering coefficient values in subjects with lighter skin versus subjects with much darker skin. The difference in L* values between those two groups is much higher than the anticipated variance in L* across different spatial regions of the forearm or palm.

### 4.2 Limitations of Homogeneous Skin Model for Calculating Tissue Scattering and Absorption from SFDI Measurements in Subjects with High Epidermal Melanin Content

At longer wavelengths, the thin superficial absorbing layer (epidermis) is expected to have less impact on the paths of NIR photons than visible-wavelength photons, so a homogeneous model of light transport is likely suitable in these circumstances. However, at shorter wavelengths, photon paths are more heavily weighted toward the epidermis, so subjects with high epidermal melanin content cannot be accurately characterized with a homogeneous tissue model in this case. Despite this issue, photon migration models for analysis of diffuse optics data often approximate the tissue as a semi-infinite homogeneous slab representing the average “bulk” tissue optical properties. Several groups have previously highlighted concerns about the application of such simple models to complex biological structures such as skin, specifically in regards to the presence of a highly localized melanin layer in the epidermis.^19^^–^^23^ For this reason, multilayered photon migration models have been developed with complex geometries to decouple the contribution of melanin from hemodynamic measurements.^24^^–^^27^ Our research on these types of layered models^28^^–^^30^ is ongoing and shows promise for addressing these issues.

Specifically, the findings of this report indicate that correlations between L*, measured $\mu_{a}$, and measured ${\mu_{s}}^{\prime }$ all contribute to the error in SFDI-based characterization of darker skin. Subjects with very low L* values are expected to have very high $\mu_{a}$ values in the epidermis. However, for the subjects with darker skin, the value of $\mu_{a}$ in the epidermis was likely significantly underestimated because the $\mu_{a}$ value from the homogeneous model is a complex weighted average of the $\mu_{a}$ values from the epidermis, dermis, and subdermal layers. This underestimation of $\mu_{a}$ likely led to a corresponding underestimation of ${\mu_{s}}^{\prime }$ for patients with darker skin tones. The large difference between the absorption coefficient of the epidermis and that of the calibration phantom may have further impacted the accuracy of optical property reconstruction for the subjects with darker skin tones; systematic investigation of this discrepancy will also be included in a future report.

It is also important to note that the quantification of skin optical properties with SFDI can be impacted by curvature, surface texture, and other anatomical heterogeneities such as hair. However, we do not anticipate that these properties provided significant confounding of the results presented in this report because we used a small ROI ($∼1\;{\mathrm{cm}}^{2}$) to avoid notable artifacts from curvature, hair, and large variations in skin texture. Surface roughness may also contribute to the measured scattering coefficient, and this contribution may indeed be different for the forearm versus the palm, so quantifying the effect of surface roughness on measured optical properties (e.g., performing SFDI measurements before and after moisturizing skin) may be worth considering as a subject of future study. Regardless, the strong correlation between L* values and SFDI-measured tissue properties is likely largely invariant with surface roughness because all subjects with low forearm L* values exhibited much lower measured reduced scattering coefficients at the lower wavelengths, so differences in surface roughness between these subjects likely did not present a significant confound.

### 4.3 Potential Impact of a Multilayered Model of Diffuse Optical Measurements of Skin

The correlation observed in this report between L* and the SFDI-measured ${\mu_{s}}^{\prime }$ of skin provides a rigorous confirmation that, for subjects with high epidermal melanin levels, SFDI-measured ${\mu_{s}}^{\prime }$ values are systematically underestimated at visible wavelengths when a homogeneous photon–tissue interaction model is used. These findings clearly demonstrate the necessity for a layered skin model that explicitly accounts for variations in epidermal melanin concentration.

Many groups have taken significant strides in developing and applying such models, based in part on simulation.^15^^,^^24^^–^^26^^,^^29^^,^^31^^,^^32^ Schmitt et al. used a layered diffusion model to simulate optical properties from a single point for a given source–detector separation.^24^ They used reference values for $\mu_{a}$ and ${\mu_{s}}^{\prime }$ from previous reports to model the epidermis, dermis, and subcutaneous layers at 660 and 950 nm. They validated their model on tissue-simulating phantoms and forearm skin in Caucasian and African American subjects. Fredriksson et al. used an inverse Monte Carlo technique to model the epidermis, superficial dermis, and more vascularized dermis using measurements from both diffuse reflectance spectroscopy (DRS) and laser Doppler flowmetry over a wavelength range of 450 to 850 nm.^26^ This model was validated *in vivo* in a large cohort of Swedish subjects.^15^ Verdel et al. paired DRS with pulsed photothermal radiometry in a four-layer (epidermis, papillary dermis, reticular dermis, and subcutaneous) model and a Monte Carlo model for light–tissue interaction.^25^ This model, based on both referenced optical property values and optimized scattering parameters for 400 to 650 nm, was tested against tanned and untanned upper arm skin from Fitzpatrick type II patients. This report used pressure cuff occlusion data to further test the model.

Spatial frequency domain spectroscopy techniques, a point-based form of SFDI, have also been analyzed with layered tissue models. Horan et al. detailed a method for measuring optical properties in a two-layered tissue-simulating phantom using an N’th-order spherical harmonic expansion with Fourier decomposition forward solver over a 450 to 1000 nm wavelength range.^32^ Saager et al. used simulated optical property values generated through Monte Carlo modeling to build a two-layer model.^31^ The model was validated using tissue phantoms with varying thicknesses of the superficial layer. This model was also validated on a series of subjects during venous and arterial occlusions.^28^ The cohort of subjects used in our report may provide a basis to develop similar models that are based on patient data from subjects with a wide range of skin tones. In fact, we are currently in the process of developing a layered skin model specifically designed to accurately fit SFDI-measured diffuse reflectance data from subjects with darker skin tones.^30^ Comprehensive validation of this model, including the crucial step of direct quantitative comparison between the layered and homogeneous models, is beyond the scope of this paper and will instead be the topic of a subsequent report. It may also be possible to use a linear regression model of the relationship between L* and ${\mu_{s}}^{\prime }$ to correct the measured ${\mu_{s}}^{\prime }$ values of subjects with darker skin tones; this topic is a subject of ongoing research by our group.

## 5 Conclusion

In this report, we show that, in subjects with dark skin tones, SFDI measurements of tissue absorption and scattering coefficients correlate strongly with the L* parameter obtained via colorimetry when a homogeneous model of photon–tissue interaction is employed. This phenomenon is particularly prominent at shorter visible wavelengths, where epidermal melanin contributes greatly to tissue absorption. These findings rigorously characterize the manner in which diffuse optics data analysis algorithms designed for light skin can provide systematic inaccuracies in tissue property measurements for patients with dark skin, even when a quantitative technique such as SFDI is employed for data acquisition.

## Code, Data, and Materials Availability

Datasets and analysis codes used in this manuscript are available upon email request to Professor Anthony J. Durkin and Professor Robert H. Wilson.
